# Organic acid metabolism in Chinese dwarf cherry [*Cerasus humilis* (Bge.) Sok.] is controlled by a complex gene regulatory network

**DOI:** 10.3389/fpls.2022.982112

**Published:** 2022-09-02

**Authors:** Caizhen Guo, Pengfei Wang, Jiancheng Zhang, Xiwen Guo, Xiaopeng Mu, Junjie Du

**Affiliations:** ^1^College of Horticulture, Shanxi Agricultural University, Jinzhong, China; ^2^Department of Life Sciences, Luliang University, Luliang, China

**Keywords:** Chinese dwarf cherry, organic acids, transcriptome, differentially expressed genes, weighted gene co-expression network association analysis

## Abstract

The acidity of Chinese dwarf cherry [*Cerasus humilis* (Bge.) Sok.] fruits is a key factor affecting the sensory quality of fruits, and it undergoes great changes during development. The molecular mechanisms of these changes are still unclear. In this study, fruits of high-acid ‘Nongda4’ and low-acid ‘DS-1’ varieties of Chinese dwarf cherry were used to determine the acid content at different developmental stages. We used transcriptome profiles to identify key genes related to organic acid metabolism and construct their co-expression networks, and we studied the expression patterns of key genes in 36 Chinese dwarf cherry accessions. The titratable acid content of both ‘DS-1’ and ‘Nongda4’ fruits first increased and then decreased during fruit development; however, the titratable acid content of ‘DS-1’ fruits changed to a minor extent. The organic acid content of ‘Nongda4’ was significantly higher than that of ‘DS-1’. The organic acids in mature fruits were mainly malic acid and citric acid. Analysis of the differentially expressed genes related to organic acid metabolism revealed six key genes, including two MDH genes, one tDT gene, one ME gene, one PEPCK gene, and one VHA gene. Weighted gene co-expression network association analysis revealed four modules that were significantly correlated with organic acid content, and 10 key genes with high connectivity among these four modules were screened, including two PK genes, two MDH genes, two ME genes, one PEPCK gene, one VHA gene, one PEPC gene, and one tDT gene. According to the expression patterns of genes in different Chinese dwarf cherry accessions, seven genes were confirmed to represent key genes related to the regulation of organic acids during Chinese dwarf cherry fruit development. These results provide a foundation for further studies on the molecular mechanism of organic acid accumulation in Chinese dwarf cherry fruit.

## Introduction

Acidity is an important factor that affects the sensory quality of fruits and also helps determine the harvest time (Jiang et al., 2019). Fruit acidity is influenced by the types and contents of organic acids, which differ among species, developmental stages, and tissues (Zhang et al., 2010; Mu et al., 2018). However, the organic acids in most fruits mainly comprise malic acid and citric acid (Zheng et al., 2020). For example, the main organic acid in ‘Honey Crisp’ apple is malic acid, the content of which first increases and then decreases with the development of the fruit; however, the overall content increases (Li et al., 2014). In contrast, the main organic acid in sand pear fruit is also malic acid, although the organic acid content decreases with the development of the fruit (Du et al., 2012).

The metabolic process of organic acids in plants is very complex, and their synthesis and transformation are completed in fruits. The components of organic acids are synthesized by various enzymes in the cytoplasm and mitochondria of flesh cells. The enzymes involved in metabolism mainly include malate dehydrogenase (MDH), malic enzyme (ME), and phosphoenolpyruvate carboxylate phosphoenolpyruvate carboxylase (PEPC). Most of the synthesized organic acids are stored in the vacuoles as vacuolar membrane H^+^-ATPase (VHA) pumps H^+^ into the vacuole to form a positive potential difference, which provides a driving force for the subsequent transport of organic acids by the vacuolar membrane dicarboxylic acid transporter (tDT) (Terrier et al., 1998; Zhang et al., 2019). Enzymes and genes involved in organic acid metabolism and storage can regulate acidity changes during fruit development (Roongruangsri et al., 2012). Consequently, it is essential to study the expression of genes related to acid metabolism during fruit development. In many fruits, including peach (Zheng et al., 2020), plum (Wang, 2015), tomato (Guillet et al., 2002), grape (Lin et al., 2020), and lemon (Sadka et al., 2000), organic acid production has been found to be regulated by related enzymes during fruit development; however, there are few differences in their regulatory mechanisms.

Chinese dwarf cherry [*Cerasus humilis* (Bge.) Sok.] is a unique shrub and fruit tree in China (Fu et al., 2021). Chinese dwarf cherry fruits have high nutrition levels, high resistance, and high health benefits(Fu et al., 2021). The cherries are rich in calcium, protein, vitamins, organic acids, and other nutrients (Mu et al., 2015; Wang et al., 2018), and they have broad research and utilization prospects. Our team previously investigated the titratable acid (TA) content in different varieties of Chinese dwarf cherry fruits and found that the acidity in Chinese dwarf fruits varies greatly across different varieties and development stages (Wang et al., 2011; Ye L. et al., 2017). At present, most of the Chinese dwarf cherry fruits cultivated have high acidity and are mostly used for preparing fruit juice and wine as few varieties with low acidity are consumed as fresh food (Wang, 2015), which greatly limits the industrialization of Chinese dwarf cherry. Therefore, the mechanisms of regulation of fruit organic acid contents have been researched extensively, although the gene regulatory network and molecular mechanisms of organic acid metabolism in Chinese dwarf cherry fruits have not been studied. Therefore, in this study, the high-acid variety ‘Nongda4’ and low-acid variety ‘DS-1’ of Chinese dwarf cherry were used to investigate the composition and content of acid in the fruits at different developmental stages. Subsequently, the differentially expressed genes (DEGs) related to organic acid metabolism in each developmental stage were analyzed via transcriptome sequencing technology, and the weighted gene co-expression network association analysis (WGCNA) method was used to screen for key genes related to organic acid metabolism in Chinese dwarf cherry fruits and construct their gene regulatory network. The results of this study will provide a molecular biological basis for the differences in organic acid contents in Chinese dwarf cherry fruits.

## Materials and methods

### Plant materials

**FIGURE 1:**
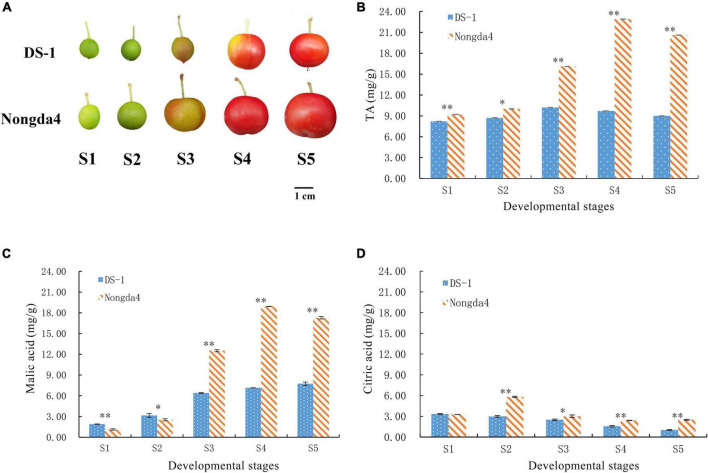
Photograph of Chinese dwarf cherry fruits at different developmental stages **(A)**. S1, young fruit stage; S2, hard core stage; S3, turning stage; S4, coloring stage; S5, mature stage. Changes in TA (titratable acid) **(B)**, malic acid **(C)**, and citric acid **(D)** contents in Chinese dwarf cherry fruits during development. Data are means ± standard deviations of three biological repetitions. Error bars represent standard deviations of means. * and ^**^ indicate significant differences at *P* < 0.05 and <0.01 levels.

‘Nongda4’ and ‘DS-1’ Chinese dwarf cherry fruits were obtained from the germplasm nursery of Shanxi Agricultural University, Jinzhong, China (37°230 N, 112°290 E). Fruit samples of each variety were collected at five developmental stages: young fruit stage (S1), hard core stage (S2), turning stage (S3), coloring stage (S4), and mature stage (S5) (Figure 1A). Following collection, the fruit samples were stored at –80°C for later analyses.

### Determination of titratable acid, malic acid, and citric acid contents

Determination of TA content was performed using the sodium hydroxide titration method (Cao et al., 2007). For sampling, flesh parts of the fruits were mixed, ground, and then weighed for the test. Extraction and determination of malic acid and citric acid content was performed using the method described by Zhang et al. (2019). Briefly, 1.0 g of fruit sample was weighed, to which 6 ml 80% ethanol solution was added following extraction at 35°C for 20 min, and centrifugation at 4,000 × *g* for 15 min, after which the supernatant was collected. The supernatant was dried using a rotary evaporator at 70°C, and dissolved in ultrapure water. The sample solution was filtered through a 0.45-μm microporous membrane, and the filtrate was used for ultra-performance liquid chromatography (UPLC) analysis. Three replicates of each sample were analyzed.

### Transcriptome sequencing and alignment with reference genome

Total RNA from the fruits of ‘DS-1’ and ‘Nongda4’ varieties at five developmental stages was extracted using Trizol reagent (15596018; Thermo Fisher Scientific, Waltham, MA, United States) following the manufacturer’s protocol. Subsequently, Lianchuan Biological Company was commissioned for quality control for total RNA, cDNA library construction, and sequencing. We aligned reads of all samples to the Chinese dwarf cherry reference genome^1^ using HISAT2 package (Yuan et al., 2022), and the number of reads compared to the reference genome was counted. According to the comparison results of HISAT2, the expression levels of all genes in each sample were calculated using the fragments per kilobase of transcript per million mapped reads (FPKM) value as the measurement index of gene expression level.

### Search for genes related to organic acid metabolism and identification of differentially expressed genes

Based on the overall annotation results of transcriptome sequencing and genes predicted in this analysis, the genes encoding enzymes related to organic acid metabolic pathways in Chinese dwarf cherry fruits were screened and analyzed for differential expression. Differential expression analysis was performed using DESeq2 software between two different groups (Liu et al., 2021). Genes a false discovery rate <0.05 and absolute fold change ≥2 were considered DEGs.

### Weighted gene co-expression network association analysis

Weighted gene co-expression network association analysis was performed in R using default parameters (Li et al., 2022). The WGCNA package associated the phenotype data with the classified modules. The organic acid content and gene expression (FPKM value) of all samples were used as input files for WGCNA analysis. The core genes in organic acid biosynthesis pathways were screened and a regulatory network was constructed. The co-expression modules of the core genes were visualized using Cytoscape 3.8.2 (Liu et al., 2021).

### Quantitative reverse transcription-polymerase chain reaction validation for transcriptome data

To verify the reliability of the RNA sequencing (RNA-Seq) data, the expression levels of nine DEGs were detected using quantitative reverse transcription-polymerase chain reaction (qRT-***P***CR), including seven key genes and two transcription factors related to organic acid metabolism. Fruit RNA was extracted using the CTAB method, cDNA was synthesized using a PrimeScript™ RT Reagent Kit (Takara, Tokyo, Japan), and then qRT-PCR analysis was performed. qRT-PCR primers were designed using the NCBI website (Table 1). The relative expression values were calculated using the 2^–ΔΔ^ ^Ct^ method (Liu et al., 2021), and the correlation was analyzed using SAS software (Liu et al., 2021).

**TABLE 1:** Expression primers for qRT-PCR.

Gene ID	Primer (5′→3′)	
	Forward	Reverse
Actin	GCAGCGACTGAAGACATACAAG	GGTGGCATTAGCAAGTTCCTC
MSTRG.20567	ACATAATGCTGGCCGGTTTC	AGCATCCGCCTCAACAAATC
MSTRG.1941	AGGCTGTGGCTGATAACTGT	AACTTCTGCAGCAATTGGCA
MSTRG.15150	TGTGGAAGCTAAGGCTGGAA	CCCTTCAGACAAGCATCAGC
MSTRG.13863	CGCCCACGTGGAATGTATTT	GACTGCCATCTGTCAGGACT
MSTRG.276	AATGCTGCCAGAAGTGCAAA	CCAGACGCTTCACATTAGCC
MSTRG.4608	GTGCTGGTGATGGAACTGTC	AAATGTCTGCAAGGCCACTG
MSTRG.5073	TGCTGATGCTCTTCGTGAGG	GCCTTCAATTCGTCCAGCAC
MSTRG.15924	GGGTTCTGTGCAGCATTCAA	TTCTTCGAACAGTGGCATCG
MSTRG.2821	GCTGTCTCTGGATCTCTCAGTC	GTGCCACAGTGGTAGATAGGG

### Functional validation of candidate genes in different Chinese dwarf cherry accessions

To further verify the function of key genes, seven key structural genes related to organic acid metabolism were selected and the expression profiles of 36 Chinese dwarf cherry accessions (Supplementary Table 1) were investigated. Gene expression analysis using qRT-PCR was performed using three independent biological replicates for each sample. Primer design and qRT-PCR were performed as described previously.

## Results

### Determination of titratable acid, malic acid, and citric acid contents

The TA content in ‘DS-1’ and ‘Nongda4’ fruits first increased and then decreased with the development of Chinese dwarf cherry fruits (Figure 1B). The TA content of ‘DS-1’ increased by 24.39% from the stage S1 (8.2 mg/g) to S3 (10.2 mg/g) and then slowly decreased by 11.76% at stage S5 (9 mg/g). The TA content varied to a minor extent during fruit development. TA content of ‘Nongda4’ increased by 148.91% from stage S1 (9.2 mg/g) to S4 (22.8 mg/g) and slightly decreased by 10.04% at stage S5 (20.6 mg/g). The TA content increased considerably and then decreased to a minor extent during the entire fruit development process. The TA content in ‘Nongda4’ fruits was always considerably higher than that in ‘DS-1’ fruits.

Malic acid and citric acid were the main organic acids in mature fruits of ‘DS-1’ and ‘Nongda4’, and their collective content accounted for 97.33 and 96.02% of the TA, respectively. The malic acid content of ‘DS-1’ increased from stage S1 (1.9 mg/g) to stage S5 (7.74 mg/g) with the development of fruits, accounting for 86% of the total acid content. The citric acid content decreased slowly from stage S1 (3.33 mg/g) to stage S5 (1.02 mg/g), accounting for 11.33% of total acid content. Both malic acid and citric acid contents were positively correlated with TA content (*P* < 0.01), and the malic acid and citric acid contents were negatively correlated (*P* < 0.01). This indicated that the acidity of this variety was determined by these contents. The malic acid content of ‘Nongda4’ increased from stage S1 (1.13 mg/g) to S4 (18.92 mg/g) and then decreased at stage S5 (17.29 mg/g), accounting for 83.93% of the total acid content. The citric acid content increased from stage S1 (3.26 mg/g) to S2 (5.82 mg/g) and then decreased at stage S5 (2.49 mg/g), accounting for 12.09% of the total acid content. This suggested that the acidity of this variety is mainly determined by malic acid content (Figures 1C,D and Table 2). In summary, stage S1 to stage S3 and stage S4 represented the periods of acid accumulation in ‘DS-1’ and ‘Nongda4’, respectively, which then decreased slightly at stage S5. In mature fruits, the malic acid and citric acid contents in ‘Nongda4’ fruits were 2.23- and 2.44-fold higher than that in ‘DS-1’ fruits, respectively, and both were significant. Therefore, the acidity of ‘Nongda4’ fruits was significantly higher than that of ‘DS-1’ fruits.

**TABLE 2:** Correlations of malic acid, citric acid and titratable acids.

Varieties	Sort	Total acid	Malic acid	Citric acid
DS-1	Titratable acid	–	0.982**	0.968*
	Malic acid	0.982**	–	–0.923*
	Citric acid	0.968**	–0.923**	–
Nongda4	Titratable acid	–	0.984**	–0.023
	Malic acid	0.984**	–	–0.722
	Citric acid	–0.023	–0.722	–

* and ** indicate significant differences at 0.05 and 0.01 levels.

### Transcriptome sequencing analysis

All samples of ‘DS-1’ and ‘Nongda4’ were analyzed via transcriptome sequencing. The average valid reads ratio obtained after screening of original data was 97.60%, the average Q30 percentage was 96.37%, the proportion of GC content was between 46 and 47%, which indicated that the sequencing quality was high and subsequent analysis can be performed. The clean reads of all samples were sequence-aligned to the Chinese dwarf cherry reference genome, and the alignment efficiencies ranged from 87.18 to 91.92%; the comparison rate was high (Supplementary Table 2).

### Search for genes related to organic acid metabolism and identification of DEGs

According to gene annotation data, 57 structural genes encoding 14 enzymes related to organic acid metabolic pathways were identified, including three pyruvate dehydrogenases (PDHs), two citrate synthetases (CSs), two isocitrate dehydrogenases (IDHs), one succinate dehydrogenase (SDH), five MEs, five MDHs, two PEPCs, two phosphoenolpyruvate carboxykinases (PEPCKs), eight aluminum-activated malate transporters (ALMTs), one tDT, 22 VHAs, four pyruvate kinases (PKs), and one fumaric acid (FH).

**FIGURE 2:**
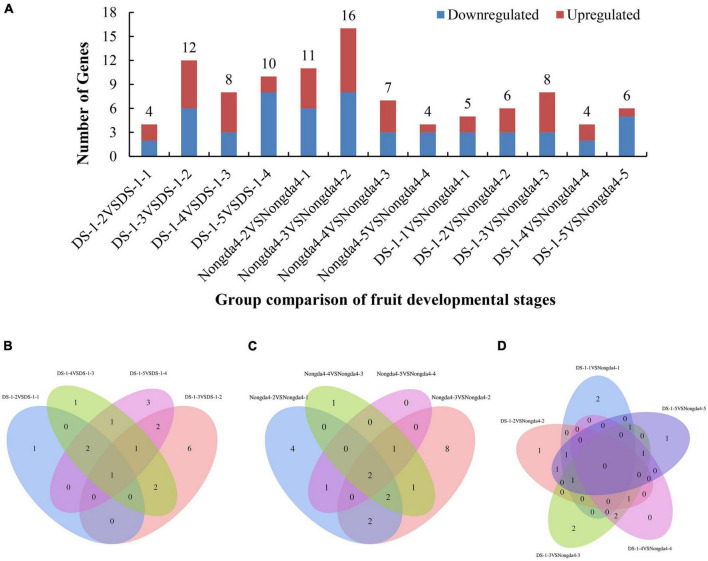
The number of differentially expressed genes related to organic acid metabolism **(A)**, and Venn diagrams illustrating differential metabolism between different developmental stages of DS-1 **(B)**, different developmental stages of Nongda4 **(C)**, two varieties in the same developmental stage **(D)**. DS-1-1, DS-1-2, DS-1-3, DS-1-4, DS-1-5, Nongda4-1, Nongda4-2, Nongda4-3, Nongda4-4, and Nongda4-5 indicate DS-1 and Nongda4 fruits at S1, S2, S3, S4, and S5 developmental stages, respectively.

To further search for DEGs related to changes in organic acid content of Chinese dwarf cherry fruits based on the organic acid content data and transcriptome sequencing results, the expression levels in all samples of ‘DS-1’ and ‘Nongda4’ were analyzed and significant DEGs were identified (Figure 2A). Both ‘DS-1’ and ‘Nongda4’ had more DEGs at the early developmental stages, and the number of total DEGs reached the highest at stage 3, suggesting that the metabolic activity was high in Chinese dwarf cherry fruits from stage S1 to S3, and stage S3 was the key stage of acid accumulation. Organic acid metabolism-related DEGs are presented in a Venn diagram in Figure 2. The four groups of ‘DS-1’ fruit developmental stages had only one common DEG and 11 unique DEGs (Figure 2B); the four groups of ‘Nongda4’ fruit developmental stages had only two common DEGs and 13 unique DEGs (Figure 2C). There were no common DEGs in both ‘DS-1’ and ‘Nongda4’ fruits; however, there were 6 unique DEGs (Figure 2D). In summary, there were more DEGs during fruit development of the same variety than between varieties, and there are both common and unique DEGs with diverse gene expression patterns. This shows that the difference in gene expression between the fruit development stages of ‘DS-1’ and ‘Nongda4’ is more significant than that between varieties.

**FIGURE 3:**
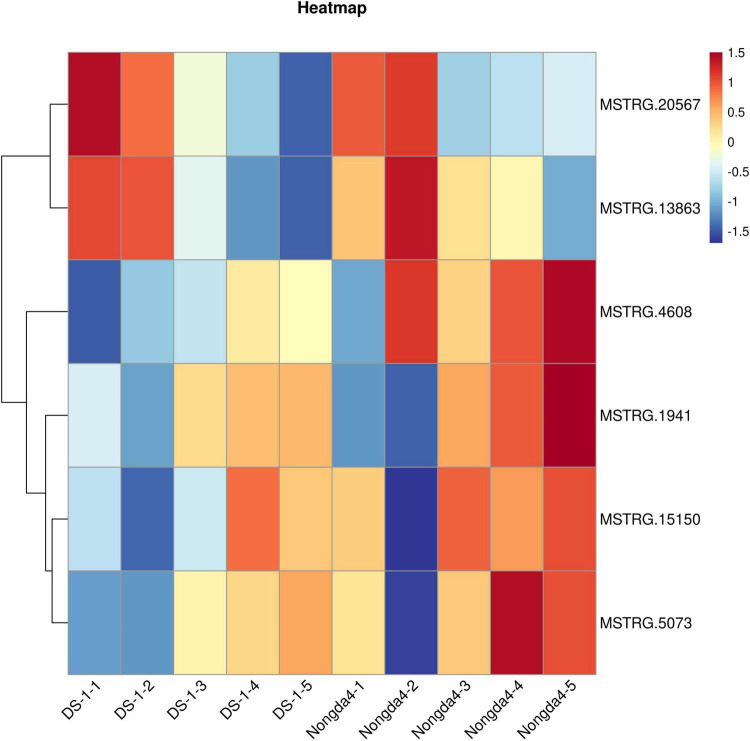
Heatmap of the expression of key genes. Red boxes represent high expression and blue boxes represent low expression. DS-1-1, DS-1-2, DS-1-3, DS-1-4, DS-1-5, Nongda4-1, Nongda4-2, Nongda4-3, Nongda4-4, and Nongda4-5 indicate DS-1 and Nongda4 fruits at S1, S2, S3, S4, and S5 developmental stages, respectively.

To further study the role of the DEGs in organic acid metabolism, their expression patterns were analyzed. The *MSTRG.1941* and *MSTRG.15150* genes encode NAD-MDHs, and *MSTRG.4608* encodes a tDT; these three genes were upregulated in ‘DS-1’ fruits with fruit development and slightly downregulated at maturity. However, the genes in ‘Nongda4’ fruits were continuously upregulated during the entire development period. The genes were positively correlated with the contents of malic acid and TA in ‘DS-1’ and ‘Nongda4’ fruits. The expression level of the genes in ‘Nongda4’ was higher than that in ‘DS-1’ fruits. Therefore, the acidity of ‘Nongda4’ fruits was higher than that of ‘DS-1’ fruits. The *MSTRG.13863* gene encodes an NAD-ME and its expression levels showed a decreasing trend with fruit development; the expression levels were negatively correlated with malic acid and TA contents in the fruits. However, the expression level in low-acid ‘DS-1’ was lower than that in high-acid ‘Nongda4’ fruits; it was speculated that the gene encoding NAD-ME that can degrade malate in ‘Nongda4’ is highly expressed, but more malic acid is accumulated. Fruit acidity was not determined by a single gene. The expression level of *MSTRG.20567*, which encodes a PEPCK, decreased with fruit development. The expression was negatively correlated with malic acid and TA contents in the fruit. *MSTRG.20567* expression in ‘Nongda4’ fruits was lower than that in ‘DS-1’ fruits. The expression level of *MSTRG.5073*, which encodes a VHA, increased continuously with fruit development. The expression was positively correlated with the malic acid and TA contents in the fruit, and the expression level in high-acid ‘Nongda4’ was higher than that in low acid ‘DS-1’. In summary, the expression patterns of *MSTRG.1941* and *MSTRG.15150* (NAD-MDH), *MSTRG.4608* (TDT), *MSTRG.13863* (NAD-ME), *MSTRG.20567* (PEPCK), and *MSTRG.5073* (VHA) were consistent with the trends of organic acid content (Figure 3). These results indicated that these genes represented the key genes associated with differences in acidity between varieties and developmental stages of Chinese dwarf cherry fruit.

### Weighted gene co-expression network association analysis

**FIGURE 4:**
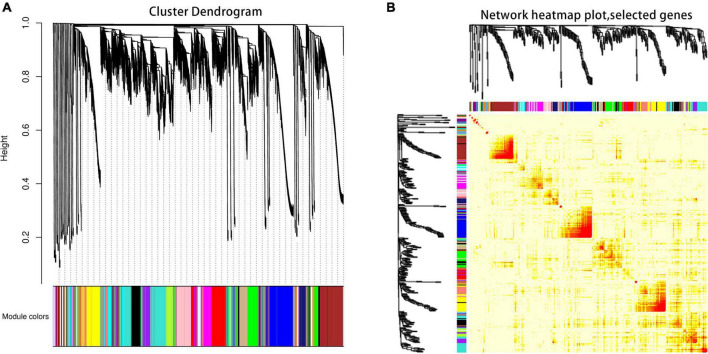
Gene module construction **(A)** and correlation analysis **(B)**.

The co-expression network of DEGs between ‘DS-1’ and ‘Nongda4’ varieties was constructed using WGCNA to yield 47 network modules and a hierarchical cluster according to different colors (Figure 4A). The number of genes in different modules varied greatly. Clustering the module transcripts and analysis of the correlation of module genes showed that the correlation between transcripts in the same module was good and the module division was reliable, which could be used for further analysis (Figure 4B).

**FIGURE 5:**
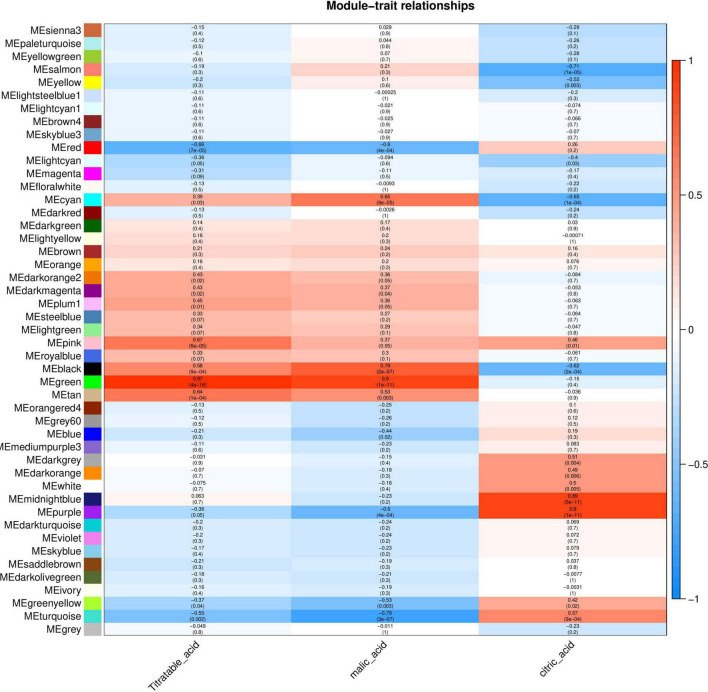
Correlation between organic acid content and modules of Chinese dwarf cherry fruit. The colors from blue to red represent r^2^ values from –1 to 1.

The relationship between each module and phenotype was quantified via Pearson correlation coefficient analysis and a heat map was generated (Figure 5). The organic acid content was significantly correlated with the Green module, and the correlation coefficient (r^2^) was 0.97. Malic acid content was significantly correlated with the Green and Turquoise modules and the correlation coefficients (r^2^) were 0.9 and –0.79, respectively. Citric acid content was significantly correlated with the Purple and Salmon modules with correlation coefficients (r^2^) of 0.9 and –0.71, respectively. These modules with high correlation represented the key modules based on all genes and connectivity information of the modules; the genes with the highest connectivity in these modules were screened as hub genes.

**FIGURE 6:**
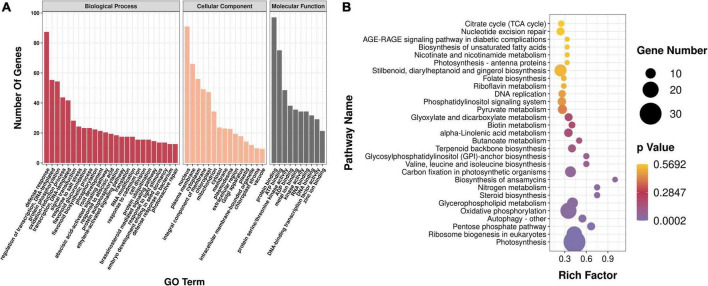
Analysis of enrichment of modules. **(A)** GO enrichment analysis. GO, Gene Ontology. **(B)** KEGG enrichment analysis. KEGG, Kyoto Encyclopedia of Genes and Genomes.

To determine the biological function of genes in each co-expression module, all the genes in the aforementioned modules were enriched and analyzed. Gene Ontology (GO) enrichment analysis showed that all genes of the module were mainly enriched in protein phosphorylation (GO:0006468), redoxidation (GO:0055114), vacuole (GO:0005773), components of plasma membrane (GO:0005887), cytoplasm (0005829), and other processes, and 11 genes related to organic acid metabolism pathway were screened (Figure 6A). Kyoto Encyclopedia of Genes and Genomes enrichment analysis showed that all genes in the module were annotated to 112 metabolic pathways; there were 59 genes in the metabolic pathway of plant pathogen interaction, followed by 30 genes in photosynthesis, 20 genes in oxidative phosphorylation, and 6 genes in organic acid metabolism pathways (Figure 6B).

**FIGURE 7:**
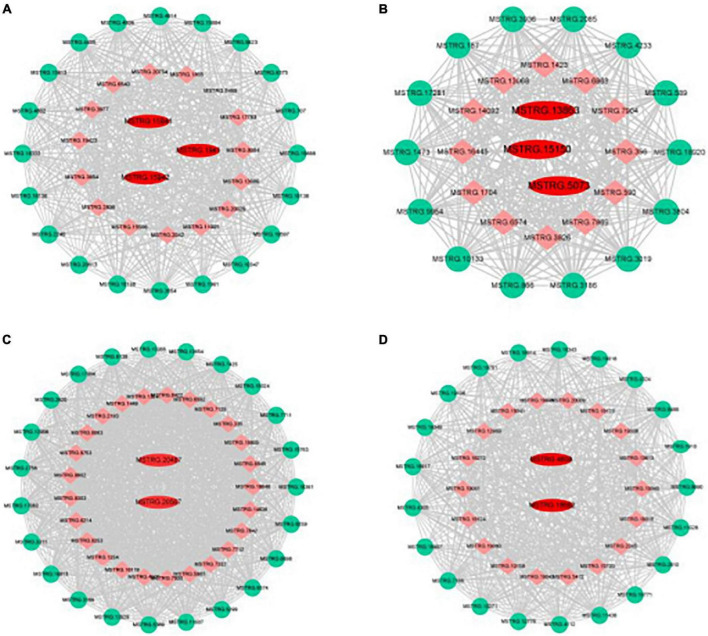
Gene co-expression networks and hub genes of **(A)** green, **(B)** purple, **(C)** turquoise, and **(D)** salmon modules. Red, hub gene; Green, transcription factor; Orange, Other genes.

Genes with high connectivity and related to organic acid metabolism in the Green, Purple, Turquoise, and Salmon modules were selected as hub genes, and the interaction network of core genes was visualized using Cytoscape software (Figure 7). The hub genes in the Green module were *MSTRG.15941*, *MSTRG.15942*, and *MSTRG.1941*; *MSTRG.15941* and *MSTRG.15942* encoded PKs and were linked to 467 and 584 genes, respectively. *MSTRG.1941* encoded an NAD-MDH and was linked to 379 genes. The hub genes in the Purple module were *MSTRG.13863*, *MSTRG.15150*, and *MSTRG.5073*; *MSTRG.13863* encoded an NAD-ME and was linked to 544 genes; *MSTRG.15150* encoded an NAD-MDH and was linked to 330 genes; *MSTRG.5073* encoded a VHA and was linked to 509 genes. The hub genes in the Turquoise module were *MSTRG.20567* and *MSTRG.20487*; *MSTRG.20567* encoded a PEPCK and was linked to 1,483 genes; *MSTRG.20487* encoded a VHA and was linked to 1,194 genes. The hub genes in the Salmon module were *MSTRG.18662* and *MSTRG.4608*; *MSTRG.18662* encoded a PEPC and was linked to 311 genes; *MSTRG.4608* encoded a tDT and was linked to 82 genes. These 10 core genes included 6 key genes screened from DEG analysis that were significantly associated with the changes in organic acid content, which indicated that core genes can be comprehensively identified using the WGCNA method.

In these modules, both interacting structural genes and transcription factors were connected with the core genes, which together constituted a complex network regulation system. Further analysis revealed that 2,127 genes interacting with the core genes were predicted to be transcription factors, belonging to 54 transcription factor families. The top transcription factor families are shown in Supplementary Figure 1. The transcription factor family containing the most DEGs was bHLH (12.23%), followed by NAC (6.58%), MYB-related (5.92%), WRKY (4.09%), and MYB (3.11%). These transcription factors may play an important role in the regulation of genes related to organic acid metabolism.

### Quantitative reverse transcription-polymerase chain reaction validation of transcriptome data

**FIGURE 8:**
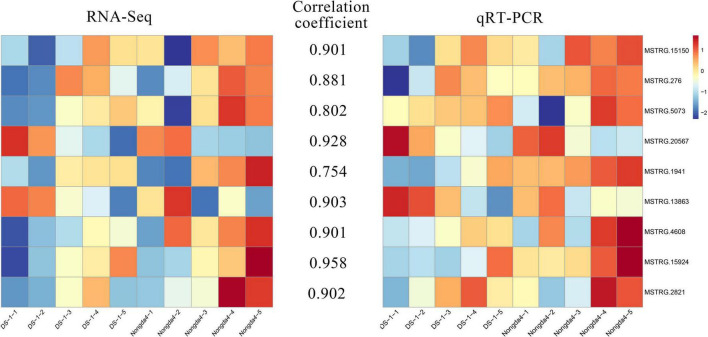
qRT-PCR validation for transcriptome data. DS-1-1, DS-1-2, DS-1-3, DS-1-4, DS-1-5, Nongda4-1, Nongda4-2, Nongda4-3, Nongda4-4, and Nongda4-5 indicate DS-1 and Nongda4 fruits at S1, S2, S3, S4, and S5 developmental stages, respectively. qRT-PCR, quantitative reverse transcription-polymerase chain reaction; RNA-Seq, RNA sequencing.

To verify the reliability of the RNA-Seq data, the expression levels of nine DEGs related to organic acid metabolism were determined via qRT-PCR (Figure 8). The relative expression levels of these genes were different in the fruits of different varieties and different developmental stages. The gene expression patterns determined via qRT-PCR and RNA-Seq were similar and the data showed a positive correlation. The genes that played a key role in the accumulation and metabolism of organic acids in Chinese dwarf cherry represented reliable indicators of acidity.

### Functional validation of candidate genes in different chinese dwarf cherry accessions

**FIGURE 9:**
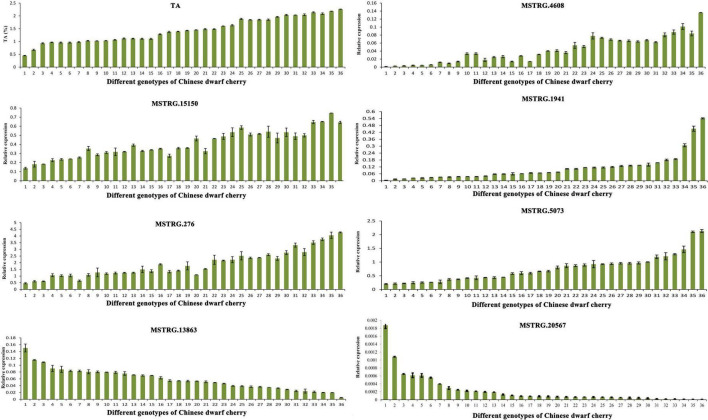
Determination of TA content and qRT-PCR expression of key genes related to organic acid metabolism in fruits of 36 Chinese dwarf cherry genotypes. TA, titratable acid; qRT-PCR, quantitative reverse transcription-polymerase chain reaction.

In total, 36 genotypes (Supplementary Table 1) were selected to investigate the expression patterns of seven key genes related to organic acid metabolism in different types of Chinese dwarf cherry plants. The TA content varied among these genotypes of Chinese dwarf cherry. The expression levels of *MSTRG.4608* (tDT), *MSTRG.15150* and *MSTRG.1941* (NAD-MDH), and *MSTRG.276* and *MSTRG.5073* (VHA) were relatively higher in accessions with high TA values. The expression levels of *MSTRG.13863* (NAD-ME) and *MSTRG.20567* (PEPCK) were relatively higher in accessions with low TA values (Figure 9). Thus, our results confirmed that these seven genes represented key genes related to organic acid regulation during Chinese dwarf cherry fruit development.

## Discussion

Organic acid metabolism has a significant correlation with fruit acidity, which is not only important in balancing fruit flavor, but also plays an important role in photosynthesis, respiration, and other metabolic processes. Organic acid metabolism involves complex biochemical pathways, and the genes in the pathways do not function alone, but are co-expressed to regulate the metabolism of organic acids. The WGCNA method can screen for core genes and construct gene co-expression networks. In the present study, we used two methods of DEG analysis related to organic acid metabolism and WGCNA in Chinese dwarf cherry fruits, and combined the data with metabolite data to identify the key genes and their regulatory networks that influence organic acid metabolism.

The contents and compositions of organic acids in fruits influence the formation of flavor. Generally, organic acids gradually accumulate with fruit development, and decrease when fruits mature owing to participation in the tricarboxylic acid cycle, glycolysis, and gluconeogenesis. The present study found that the TA content in ‘DS-1’ and ‘Nongda4’ fruits first increased and then decreased, which was similar to the change in plum fruit acidity (Singh et al., 2009). However, the organic acid content in ‘Nongda4’ fruits was significantly higher than that in ‘DS-1’ fruits during the entire fruit development process. The main organic acid in mature fruits of the two varieties was malic acid, which had a significant positive correlation with TA content, followed by citric acid. Both ‘DS-1’ and ‘Nongda4’ were malic acid-type fruits, and fruit acidity was mainly determined by malic acid and citric acid contents, which was consistent with previous findings (Wang et al., 2011; Ye L. et al., 2017). ‘Nongda4’ fruits showed relatively higher acid accumulation and lower degradation, and the accumulation and degradation of acids in ‘DS-1’ fruits changed to a minor extent. Therefore, the acidity of ‘Nongda4’ mature fruits was significantly higher than that of ‘DS-1’ fruits. The changing trend of acidity during fruit development may be related to the balance in acid metabolism (Wu and Chen, 2016). Apricots (Xi et al., 2016) and European sweet cherries (Girard and Kopp, 1998) show similar trends.

In this study, we analyzed the DEGs in Chinese dwarf cherry fruits and found that the expression levels of genes encoding NAD-MDH, tDT, and VHA were positively correlated with the malic acid and TA contents, while the expression levels of PEPCK and NAD-ME were negatively correlated with malic acid and TA contents. These genes represented key factors involved in fruit organic acid metabolism, which may explain the higher acidity of ‘Nongda4’ fruits than that of ‘DS-1’ fruits. Sweetman et al. (Yao et al., 2011) found that MDH can catalyze the mutual conversion of malic acid and oxaloacetate, which may be a key step in the synthesis of malic acid. The results of the present study are consistent with previous findings on the regulation of malic acid synthesis by MDH in peaches (Zheng et al., 2020) and Chinese dwarf cherry fruits (Wang, 2015). Malate degradation is mainly regulated by ME, and Ling et al. found that the ME-encoding gene in apples negatively regulates the accumulation of malic acid during fruit development(Walker et al., 2015), and the same results have been found in pears (Li et al., 2013) and loquats (Chen et al., 2009). PEPCK catalyzes the conversion of oxaloacetate to phosphoenolpyruvic acid and carbon dioxide. PEPCK mainly plays a role in malic acid catabolism in grapes, tomatoes, blueberries, and other fruits (Famiani et al., 2005). Terrier et al. (1998) found an AttDT homologous gene in grapefruit and confirmed that it participates in the transport of malic acid across the vacuolar membrane and positively affects accumulation of malic acid. Yao et al. (2010) found that VHA gene expression is positively correlated with malic acid content in fruits. Therefore, the findings of the present study were consistent with previous findings. The present study provides sufficient data for follow-up studies on the regulation of organic acid metabolism, and provides a foundation for further mining of key genes and transcription factors.

**FIGURE 10:**
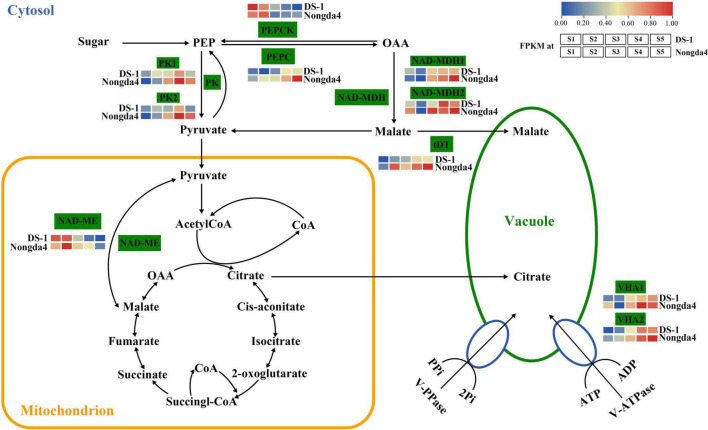
The organic acid biosynthetic pathway Enzyme names are shown along with their expression patterns at various stages. Grids represent the expression patterns of genes shown as FPKM values: S1, S2, S3, S4, and S5, left to right. NAD-ME, NAD-malic enzyme; NAD-MDH, NAD-malate dehydrogenase; PEPCK, phosphoenolpyruvate carboxykinase; VHA, V-type ATPase; PEPC, phosphoenolpyruvate carboxylase; PK, pyruvate kinase; tDT, tonoplast dicarboxylate transporter.

Traditional gene differential expression analysis only focuses on the function of a single gene, thereby ignoring the interaction between genes with similar expression patterns. In contrast, WGCNA can simultaneously compare gene expression of a large number of samples and identify co-expressed genes by clustering genes, which are highly correlated at the transcriptional level, into various modules related to biological properties. Therefore, WGCNA enables comprehensive analysis and is suitable for processing complex large-scale gene expression data (Zhao et al., 2010). In the present study, 10 core genes related to organic acid metabolism were screened using the WGCNA method, which were *MSTRG.15941* and *MSTRG.15942* (PK), *MSTRG.1941* and *MSTRG.15150* (NAD-MDH), *MSTRG.13863* (NAD-ME), *MSTRG.5073* (VHA), *MSTRG.20567* (PEPCK), *MSTRG.20487* (VHA), *MSTRG.18662* (PEPC), and *MSTRG.4608* (tDT); the proposed roles of these genes are shown in Figure 10. These 10 hub genes contain six key genes screened via DEG analysis of the transcriptome that were significantly associated with organic acid content. The common genes screened by the two methods can be considered as key genes for follow-up research.

In the WGCNA network, the hub genes are connected with a large number of genes, indicating that the organic acid metabolism is not regulated by a single gene, but rather in cooperation with a several structural genes and transcription factors. Transcription factors, as regulatory proteins, play an important role in organic acid metabolism. Many transcription factors related to organic acid metabolism were also screened via WGCNA, and the top five transcription factor families were bHLH (12.23%), NAC (6.58%), MYB-related (5.92%), WRKY (4.09%), and MYB (3.11%). WRKY and MYB transcription factors are reported to function as regulatory factors of genes related to organic acid metabolism. Hu et al. (2016, 2017) confirmed that MDMYB1/10 affects malic acid accumulation in apples by regulating MdVHA-B1 and MdVHA-B2 expression. MdMYB73 directly activates MdALMT9, and MdVHA-A to promote the synthesis of malic acid. Ye J. et al. (2017) confirmed that SlWRKY42 can directly bind to the promoter of the *S1ALMT9* gene to negatively regulate the accumulation of malic acid in tomato fruits. However, the function of these genes in organic acid metabolism of Chinese dwarf cherry has not been reported. In addition, it is speculated that other transcription factors with high connectivity in the regulatory network may have certain functions in organic acid metabolism of Chinese dwarf cherries, and their functions should be studied further via molecular biology and biochemical tests.

## Conclusion

We studied the organic acid contents in high-acid ‘Nongda4’ and low-acid ‘DS-1’ varieties of Chinese dwarf cherry at different developmental stages, and the key genes related to organic acid metabolism were screened using transcriptome-based DEG and WGCNA methods. According to the results of the analysis of the DEGs related to organic acid metabolism in the transcriptome of Chinese dwarf cherry fruit, six key genes were identified, including two NAD-MDH genes, one tDT gene, one NAD-ME gene, one PEPCK gene, and one VHA gene; 10 key genes were screened via WGCNA, including two PK genes, two NAD-MDH genes, two NAD-ME genes, one PEPCK gene, one VHA gene, one PEPC gene, and one tDT gene. A complex regulatory network was constructed based on the 10 key genes. qRT-PCR and RNA-Seq analysis results of genes related to organic acid metabolism were consistent. The expression profiles of key genes in the genetically diverse Chinese dwarf cherry accessions further confirmed our results. Overall, the results of the present study provide a molecular biological basis for further studies on fruit organic acids regulation and could facilitate the breeding of Chinese dwarf cherry varieties with low fruit acidity in future.

## Data availability statement

The original contributions presented in this study are available in the National Center for Biotechnology Information (NCBI) BioProject database under accession number: PRJNA870305.

## Author contributions

CG: investigation and writing—original draft. PW, JZ, and JD: methodology. CG and XG: software. JD and XM: writing—review and editing. All authors have contributed to the study.

## Footnotes

1
https://figshare.com/articles/dataset/Assembled_Genome_Sequences_of_Cerasus_humilis/11669673
