# Supplementary material for: Organic acid metabolism in Chinese dwarf cherry [Cerasus humilis (Bge.) Sok.] is controlled by a complex gene regulatory network
Source: Front Plant Sci. 2022 Sep 2;13:982112. doi: 10.3389/fpls.2022.982112 (PMC9491322; doi:10.3389/fpls.2022.982112)

Supplementary Material

# Supplementary Table

**Supplementary Table 1 Chinese dwarf cherry accessions**

| Accession number | Accession |
| --- | --- |
| 1 | 3-20-7 |
| 2 | 11-2-41 |
| 3 | 1-1-5-5 |
| 4 | 01-01 |
| 5 | Jiou3 |
| 6 | C510-01 |
| 7 | 10-06 |
| 8 | DG-4 |
| 9 | Nongda7 |
| 10 | 16-10 |
| 11 | 9-2-28 |
| 12 | DG-7 |
| 13 | 10-4-3 |
| 14 | Y13-03 |
| 15 | 08-16 |
| 16 | J-2 |
| 17 | 09-01 |
| 18 | N1-14-6 |
| 19 | 11-05 |
| 20 | N1-13-2 |
| 21 | Nongda3 |
| 22 | 1-1-3-10 |
| 23 | C57-8-1 |
| 24 | 99-02 |
| 25 | Nongda5 |
| 26 | 10-32 |
| 27 | Nongda6 |
| 28 | DG-4-1 |
| 29 | Jinou2 |
| 30 | Y04-26 |
| 31 | N1-16-4 |
| 32 | Jingou2 |
| 33 | Jinou3 |
| 34 | 1-1-4-3 |
| 35 | Jingou1 |
| 36 | 15-51 |

**Supplementary Table 2 Transcriptome sequencing data**

| Sample | Raw Data | | Valid Data | | Valid Ratio(reads) | Q20% | Q30% | GC content% | Mapped reads |
| --- | --- | --- | --- | --- | --- | --- | --- | --- | --- |
|  | Read | Base | Read | Base |  |  |  |  |  |
| DS_1_1_1 | 46745084 | 7.01G | 45601014 | 6.84G | 97.55 | 99.76 | 95.44 | 46.50 | 40061343(87.85%) |
| DS_1_1_2 | 47201232 | 7.08G | 46028208 | 6.90G | 97.51 | 99.78 | 95.79 | 46.50 | 40526766(88.05%) |
| DS_1_1_3 | 55917068 | 8.39G | 54557828 | 8.18G | 97.57 | 99.80 | 96.12 | 46.50 | 48098355(88.16%) |
| DS_1_2_1 | 47298852 | 7.09G | 46161786 | 6.92G | 97.60 | 99.78 | 95.94 | 46 | 41037960(88.90%) |
| DS_1_2_2 | 47429846 | 7.11G | 46248526 | 6.94G | 97.51 | 99.77 | 95.88 | 46 | 41083454(88.83%) |
| DS_1_2_3 | 44467422 | 6.67G | 43297420 | 6.49G | 97.37 | 99.79 | 96.06 | 46 | 38600052(89.15%) |
| DS_1_3_1 | 51412428 | 7.71G | 50308198 | 7.55G | 97.85 | 99.82 | 96.02 | 46 | 44888511(89.23%) |
| DS_1_3_2 | 52781362 | 7.92G | 51668266 | 7.75G | 97.89 | 99.82 | 96.07 | 46 | 46160447(89.34%) |
| DS_1_3_3 | 53077780 | 7.96G | 51944692 | 7.79G | 97.87 | 99.83 | 96.02 | 46 | 46439393(89.40%) |
| DS_1_4_1 | 53341342 | 8.00G | 52238436 | 7.84G | 97.93 | 99.88 | 96.51 | 46 | 47013435(90.00%) |
| DS_1_4_2 | 51185194 | 7.68G | 50094512 | 7.51G | 97.87 | 99.87 | 96.62 | 46 | 45085049(90.00%) |
| DS_1_4_3 | 48458814 | 7.27G | 46779728 | 7.02G | 96.54 | 99.91 | 97.06 | 46 | 42164548(90.13%) |
| DS_1_5_1 | 50998350 | 7.65G | 49401018 | 7.41G | 96.87 | 99.93 | 97.10 | 46 | 44609101(90.30%) |
| DS_1_5_2 | 45406178 | 6.81G | 44347684 | 6.65G | 97.67 | 99.90 | 96.83 | 46 | 39920790(90.02%) |
| DS_1_5_3 | 48232084 | 7.23G | 47199788 | 7.08G | 97.86 | 99.90 | 96.55 | 46 | 42271017(89.56%) |
| Nongda4_1_1 | 41867158 | 6.28G | 40844896 | 6.13G | 97.56 | 99.86 | 96.44 | 46 | 37259437(91.22%) |
| Nongda4_1_2 | 37887772 | 5.68G | 36900322 | 5.54G | 97.39 | 99.86 | 96.38 | 47 | 32169749(87.18%) |
| Nongda4_1_3 | 42550248 | 6.38G | 41386672 | 6.21G | 97.27 | 99.87 | 96.60 | 46 | 37316014(90.16%) |
| Nongda4_2_1 | 39880888 | 5.98G | 38658776 | 5.80G | 96.94 | 99.83 | 96.24 | 46.50 | 34652059(89.64%) |
| Nongda4_2_2 | 40981948 | 6.15G | 39911144 | 5.99G | 97.39 | 99.81 | 95.78 | 46 | 35860460(89.85%) |
| Nongda4_2_3 | 41868430 | 6.28G | 40790184 | 6.12G | 97.42 | 99.82 | 95.76 | 46 | 36583196(89.69%) |
| Nongda4_3_1 | 44464844 | 6.67G | 43493440 | 6.52G | 97.82 | 99.90 | 96.66 | 46 | 39531584(90.89%) |
| Nongda4_3_2 | 43204092 | 6.48G | 42201696 | 6.33G | 97.68 | 99.89 | 96.45 | 46 | 38264840(90.67%) |
| Nongda4_3_3 | 45351040 | 6.80G | 44312664 | 6.65G | 97.71 | 99.88 | 96.50 | 46 | 40176453(90.67%) |
| Nongda4_4_1 | 47665362 | 7.15G | 46625194 | 6.99G | 97.82 | 99.91 | 96.47 | 46 | 42438020(91.02%) |
| Nongda4_4_2 | 46952800 | 7.04G | 45889444 | 6.88G | 97.74 | 99.92 | 96.76 | 46 | 41697049(90.86%) |
| Nongda4_4_3 | 52407770 | 7.86G | 51192408 | 7.68G | 97.68 | 99.91 | 96.72 | 46 | 46656220(91.14%) |
| Nongda4_5_1 | 54956474 | 8.24G | 53886780 | 8.08G | 98.05 | 99.97 | 96.84 | 46 | 49532503(91.92%) |
| Nongda4_5_2 | 54659330 | 8.20G | 53514758 | 8.03G | 97.91 | 99.93 | 96.73 | 46 | 48911395(91.40%) |
| Nongda4_5_3 | 55184882 | 8.28G | 54104768 | 8.12G | 98.04 | 99.96 | 96.88 | 46 | 49155092(90.85%) |

# 2 Supplementary Figure

# **Supplementary** Figure 1 Distribution of transcription factors in modules.


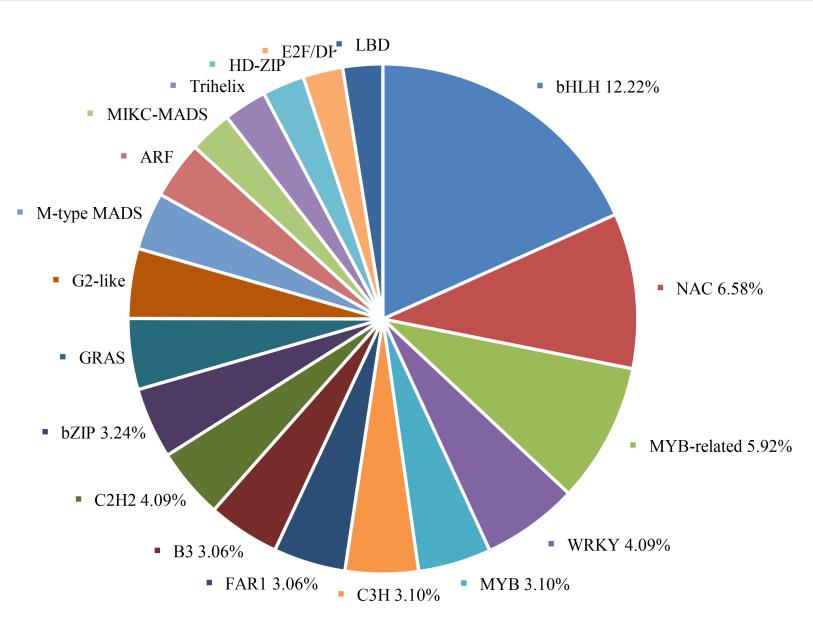

Supplement: Supplementary file 1 [file Data_Sheet_1.docx]
